# Graphene-Based THz Absorber with a Broad Band for Tuning the Absorption Rate and a Narrow Band for Tuning the Absorbing Frequency

**DOI:** 10.3390/nano9081138

**Published:** 2019-08-08

**Authors:** Qihui Zhou, Peiguo Liu, Chenxi Liu, Yuandong Zhou, Song Zha

**Affiliations:** 1 College of Electronic Science and Technology, National University of Defense Technology, Changsha 410073, China; 2 93046 Unit, China

**Keywords:** tunable absorber, graphene, terahertz device

## Abstract

In this paper, we propose a broadband absorption-controllable absorber based on nested nanostructure graphene and a narrowband frequency-tunable absorber utilizing gold-graphene hybrid structure in the terahertz regime. The numerical simulation results showed that the absorption of the broadband absorber can be changed from 27% to more than 90% over 0.75 to 1.7 THz by regulating the chemical potential of graphene. With the same regulation mechanism, the absorbing peak of the narrowband absorber can be moved from 2.29 to 2.48 THz continuously with absorption of 90%. Furthermore, via the cascade of the two types of absorbers, an independently tunable dual-band absorber is constituted. Its absorption spectrum is the superposition of absorption-controllable absorber and frequency-tunable absorber. The absorptivity and operating frequency of the two absorbing bands can be tuned independently without mutual inference. Moreover, it is insensitive to the polarization and it maintains high absorption over a wide range of incident angle. For the flexibility, tunability as well as the independence of polarization and angle, this design has wide prospects in various applications.

## 1. Introduction

In recent years, terahertz (THz) wave, lying between the microwaves and infrared light in the electromagnetic spectrum, has attracted great attention of researchers for its potential applications in communication, imaging, sensing, spectroscopy, etc. [1,2,3,4]. With the rapid development of THz technology, the demand for THz functional devices, such as filter, rotator, absorber, has become increasingly urgent [5,6,7]. Among these devices, THz absorber has drawn special concern due to its versatile utilization. However, the characteristics of absorber are determined once processed, thus limiting the scope of application. Traditional methods to realize tunability, such as diodes, varactors and liquid crystal, are no longer applicable in THz band [8]. Graphene, a monolayer of carbon atoms arranged in a honeycomb lattice, is an ideal candidate to design absorbers due to its remarkable mechanical properties, high carrier mobility, flexibility, and the ability to support localized surface plasmon resonance [9,10,11]. Moreover, the surface conductivity of graphene can be continuously tuned by regulating the chemical potential via electrostatic doping [12,13].

Hence, the research on development of tunable THz absorbers based on graphene has been expanding fast recently. To enhance the absorption and tunability, THz absorbers usually take advantage of patterned graphene, graphene-gold hybrid structure, and gold patches along with monolayer graphene [14,15,16,17]. However, the absorbers mentioned above generally have a single operating band or narrow absorption bandwidth. To overcome the mentioned drawbacks, different approaches have been proposed to achieve multiband or broadband function. The multiple bands can be realized either by integrating several resonators within a unit cell or stacking multiple layers of the resonators with different size separated by dielectric layers [18,19,20]. The broad bandwidth is implemented by adopting gradient structural elements, combining multilayer resonators, or nesting concentric resonators [21,22,23,24,25]. All of the mentioned absorbers can only regulate a single parameter in a specific design. Tuning both the absorption level and the frequency of different absorbing bands independently remains a challenging task for THz absorbers.

In this paper, we propose a dual-band THz graphene absorber with a broad absorption-controllable band and a narrow frequency-tunable band using the cascade method. Firstly, a broadband absorption-controllable absorber is designed. This absorber consists of periodic nanostructure graphene layer placed over dielectric layers backed on a reflector. The nested square ring and patch of graphene sheet achieves a high switching intensity of absorption over a wide band when adjusting the chemical potential via external gate voltage. Secondly, a narrowband frequency-tunable absorber is proposed. It is composed of a graphene-gold hybrid layer, a substrate spacer, and a gold grid, exhibiting a typical sandwich structure. The graphene strips deposited on the top layer dissipate the resonant power of gold structure, thus forming an absorption band. Besides, via controlling the external gate voltage, the resonant frequency can be regulated for the perturbation caused by the regulation of chemical potential in graphene strips. Finally, replacing the gold reflector of absorption-controllable absorber with the frequency-tunable absorber of the sandwiched structure, an independent tunable dual-band absorber is constructed.

## 2. Materials and Methods

Graphene is considered as an infinitesimally thin material, which can be modeled as a two-dimensional sheet [26,27]. The surface conductivity of graphene is contributed by inter-band and intra-band transitions according to the Kubo formula [28,29]:

(1) $$\sigma_{g}=\sigma_{g}^{intra}+\sigma_{g}^{inter}$$

(2) $$\sigma_{g}^{intra}=\frac{2k_{B}Te^{2}}{\pi \hbar^{2}}\ln\left[2\cosh\left(\frac{E_{F}}{2k_{B}T}\right)\right]\frac{i}{\omega +i\tau^{-1}}$$

(3) $$\sigma_{g}^{inter}=\frac{e^{2}}{4\hbar }\left[H\left(\omega /2\right)+i\frac{4\omega }{\pi }\int_{0}^{\infty }\frac{H\left(\Omega \right)-H\left(\omega /2\right)}{\omega^{2}-4\Omega^{2}}d\Omega \right]$$

where *k_B_* is the Boltzmann’s constant, *e* is the charge of an electron, and ℏ = *h/2π* is the reduced Planck’s constant, *E_F_* is the chemical potential (Fermi energy), *τ* is the relaxation time set as 0.1 ps, which is typical for experimentally studied graphene [30], T is room temperature set as 300 K, *H*(Ω) is defined as:

(4) $$H\left(\Omega \right)=\frac{\sinh\left(\hbar \Omega /k_{B}T\right)}{\cosh\left(E_{F}/k_{B}T\right)+\cosh\left(\hbar \Omega /k_{B}T\right)}$$

**Figure 1:**
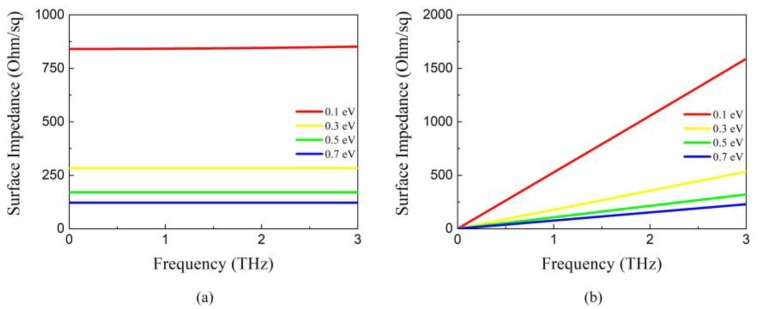
Surface impedance of graphene with different chemical potential (**a**) Real part (**b**) Imaginary part.

According to the Pauli exclusion principle, the inter-band contribution of graphene conductivity can be safely neglected in low THz region [31]. Then, the surface impedance of a graphene monolayer is calculated by *Z_g_* = 1/*σ*_g_. The real and imaginary parts of the surface impedance as a function of chemical potential is illustrated in Figure 1a,b respectively. In low THz region, the real part of the surface impedance almost remains constant, but the imaginary part grows linearly as the frequency rises. Using circuit equivalence analysis, graphene sheet behaves like the series of inductance and resistance (LR). With the increase of *E_F_*, the surface impedance decreases gradually. Therefore, the surface impedance of graphene can be effectively regulated by changing the chemical potential.

To manipulate the chemical potential of graphene via electrostatic doping, an external gate voltage is applied between the graphene layer and underneath layer. The relation between chemical potential and gate voltage can be approximately expressed by the equation [32,33]:

(5) $$E_{F}=\hbar \nu_{F}\sqrt{\pi \varepsilon_{0}\varepsilon_{r}V_{g}/ed_{s}}$$

where *v_F_* is the Fermi velocity, *ε_0_* and *ε_r_* are the permittivity of free space and the substrate between electrodes, separately. *V_g_* is the gate voltage, *e* is the electron charge, and *d_s_* is the thickness of the spacer between electrodes. Therefore, by regulating the surface impedance of graphene through the gate voltage, the intensity of surface plasmon resonance can be tuned effectively, thus controlling the absorption of THz absorber based on graphene.

**Figure 2:**
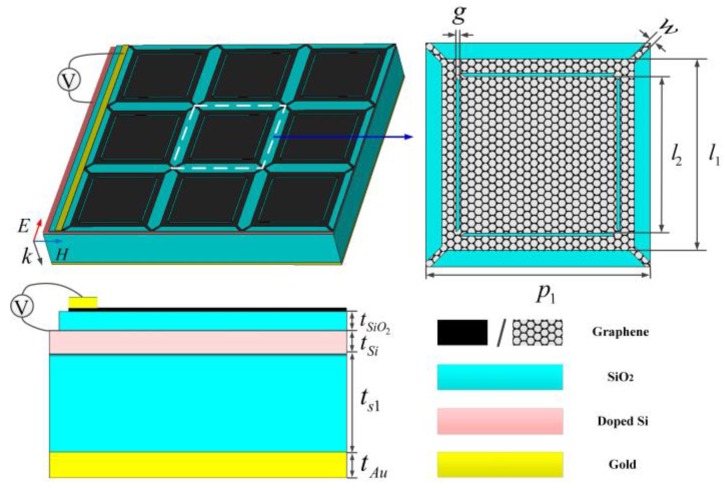
Schematic of the broadband absorption-controllable absorber. The values of the dimension parameters: *p*_1_ = 70, *l*_1_ = 60, *l*_2_ = 49, *g* = 1, *w* = 2, $t_{SiO_{2}}$ = 0.3, *t*_Si_ = 1, *t*_s1_ = 30, *t*_Au_ = 0.5, unit: μm.

Firstly, a broadband absorption-controllable absorber is proposed. Figure 2 gives the detailed structure of the broadband absorption-controllable absorber, which consists of a nanostructure graphene layer, a SiO_2_-doped Si-SiO_2_ sandwich structure, a gold ground. The nested square ring and patch of graphene can form resonance and excite localized surface plasmon resonances, which are hybridized with each other leading to a broadband absorption band [24]. The diagonals connect discrete units so that the electrostatic doping of periodic graphene array becomes much easier. The SiO_2_ layers function as spacers to separate the graphene, doped Si, and gold. It is a normal substrate and doesn’t have effects on the absorption. Doped Si plays the role of another electrode gate to realize the dynamical control of chemical potential of graphene [34,35,36]. The gold ground, whose thickness is much larger than typical skin depth in THz, reflects the incident wave like a mirror. The detailed structural parameters are listed in the caption of Figure 2. To take dispersion property of metal into consideration, the permittivity of gold is derived from Drude Model *ε*_Au_ = 1−*ω*_p_^2^/*ω*(*ω*+*iγ*_0_), where bulk plasmon frequency of gold is ω_Au_ = 1.37 × 10^16^ S^−1^ and the collision frequency is γ_0_ = 4.08 × 10^13^ S^−1^ [37,38]. The structural dimensions and thicknesses of different layers are shown in the caption of Figure 2.

**Figure 3:**
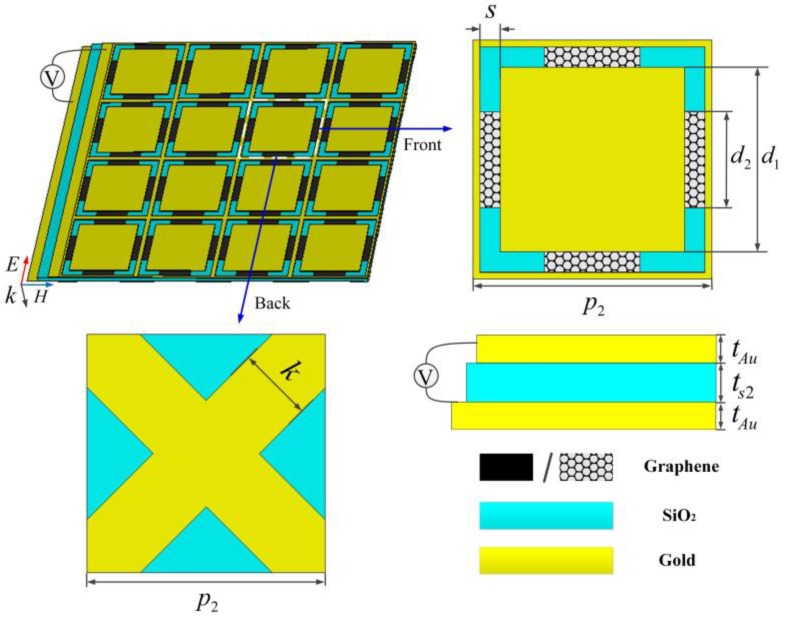
Schematic of the narrowband frequency-tunable absorber. The values of the dimension parameters: *p*_2_ = 35, *d*_1_ = 27, *d*_2_ = 14, *s* = 3, *k* = 11, *t*_Au_ = 0.5, *t*_s2_ = 2, unit: μm.

Secondly, a narrowband frequency-tunable absorber is designed. Figure 3 depicts the schematic of the THz frequency-tunable absorber, which consists of a hybrid-SiO_2_-gold sandwich structure. The front layer is a square gold patch array surrounded by the gold grid, and graphene strips are evenly deposited at the bottom of the slot. The patch-grid periodic metal structure generates a parallel capacitance and inductance (LC) resonance [39,40], whose power is dissipated by the graphene strips. The SiO_2_ acts as a spacer and also as a support layer. The back gold grid in staggered arrangement plays the role of electrode gate. As mentioned above, graphene sheet behaves like the series of inductance and resistance. By increasing the voltage between front and back layer, the inductance of graphene drops gradually, the total paralleled inductance of the resonance decreases as well, thus moving the absorbing frequency. The detailed structural parameters are shown in the caption of Figure 3.

**Figure 4:**
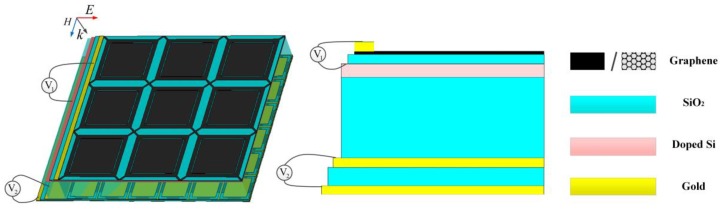
Schematic of the independently tunable dual-band absorber.

Finally, an independently tunable dual-band absorber is constituted by substituting the frequency-tunable absorber for the gold ground of the broadband absorption-controllable absorber. The absorption of the first operating band *f_1_* is controlled by the voltage between nanostructure graphene and doped Si layer *V_1_* and the frequency of the second operating band *f_2_* is tuned via the voltage between the gold-graphene hybrid layer and back grid layer *V_2_*. Since the nanostructure graphene is almost transparent to incident wave of *f_2_* and the frequency-tunable absorber reflects all of the transmitted wave of *f_1_*, two absorbing bands can be controlled individually without impacting each other. In theory, the doped Si can play electorate for both two different absorbers, but the distance between doped Si and gold-graphene hybrid structure is quite long, which may lead to an extremely high bias voltage. The introduction of cross feed network can solve this problem effectively. Besides, as shown in Figure 4, the periodicity of the patterned graphene layer is twice as large as that of the hybrid structure, so the unit cell of the integrated structure contains one unit of the top layer and four units of the bottom layer in the simulation.

## 3. Results

### 3.1. Simulations of the Broadband Absorption-Controllable Absorber

**Figure 5:**
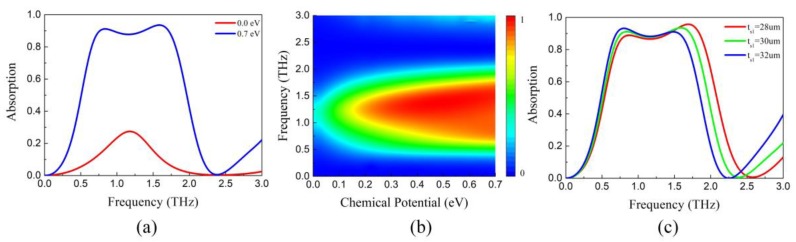
Absorption spectrum of the broadband absorption-controllable absorber (**a**) *E_F_* = 0 eV and 0.7 eV (**b**) As a function of frequency and *E_F_* (**c**) with different *t_s1_* when *E_F_* = 0.7 eV.

The full-wave simulation is conducted in the Computer Simulation Technology (CST) Microwave Studio (2015). It is a high-performance 3D EM analysis software package for designing, analyzing and optimizing electromagnetic (EM) components and systems [41]. The reflection and transmission are calculated using these equations: *R* = ǀ*S*_11_ǀ^2^ and *T* = ǀ*S*_21_ǀ^2^. Then, absorption can be obtained through *A* = 1−*R*−*T*. The influence of the chemical potential on the absorption spectrum of the broadband absorption-controllable absorber is given in Figure 5a,b. Under normal incidence, the absorption over 90% ranges from 0.75 to 1.7 THz when *E_F_* = 0.7 eV. As *E_F_* decreases from 0.7 to 0 eV, the surface impedance of graphene drops rapidly, the absorption changes continuously from 90% to 27% corresponding the intense of resonances weakened. In the process of chemical potential decline, the rate of change in absorption is gradually accelerated, consistent with that of surface impedance shown in Figure 1.

The absorption spectrum of the broadband absorption-controllable absorber with different thick spacer thickness (*t_s1_*) is shown in the Figure 5c. With the increase of *t_s1_*, the absorption curves appear a red shift. Besides, the absorptivity drops down gradually at the upper frequency while grows slowly at the lower frequency. In ground backed absorber, the thickness of substrate between patterned graphene and ground has a negative relation with the resonant frequency. Hence, the high-frequency resonance is weakened while the high-frequency one is enhanced. As a result, the operating band of the broadband absorber shifts to lower frequency with the increase of *t_s1_*.

**Figure 6:**
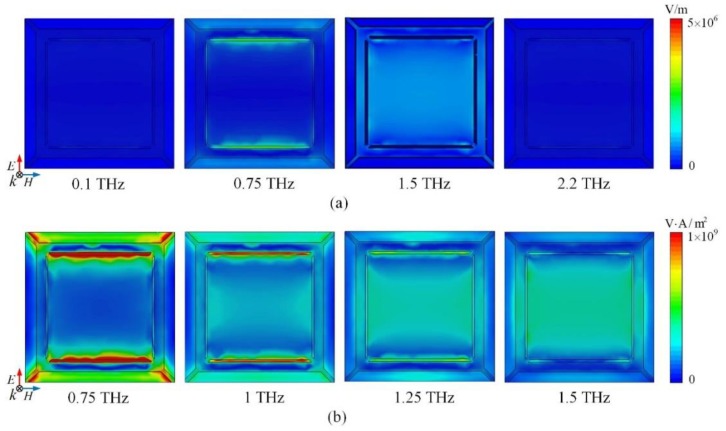
(**a**) The electric field distribution of the broadband absorption-controllable absorber at 0.1, 0.75, 1.5, 2.2 THz; (**b**) The power flow distribution of the broadband absorption-controllable absorber at 0.75, 1, 1.25, 1.5 THz.

In order to further understand the broadband absorption mechanism, the electric field distribution and power flow of the absorber with *E_F_* = 0.7 eV are investigated. Figure 6a illustrates the electric field distribution of absorber in absorbing band (0.75 and 1.5 THz) and out of absorbing band (0.1 and 2.2 THz). Obviously, the electric field at 0.1 and 2.2 THz is rather weak corresponding to low absorption. Within the absorbing band, strong electric field is concentrated on the narrow gap and adjacent units at 0.75 THz and appears in the central square patch at 1.5 THz. It means that the resonance between the ring and the patch contributes to the low-frequency absorption while localized surface plasmon resonance caused by the square graphene patch leads to the high-frequency absorption [24]. It can be further investigated by the power flow distribution displayed in Figure 6b. Since the absorption is more than 90% in the operating band, the power flow on the top layer can show the resonant intensity and power dissipation in every position. When the frequency increases from 0.75 to 1.5 THz, the power flow is gradually weakened in the gap while greatly enhanced in the central graphene patch, which agrees well with analysis of electric field. The hybridization of resonances forms the broadband absorption. And the absorption band is decided by the dimensions of the patterned graphene layer and distance between graphene and gold ground.

### 3.2. Simulations of The Narrowband Frequency-Tunable Absorber

**Figure 7:**
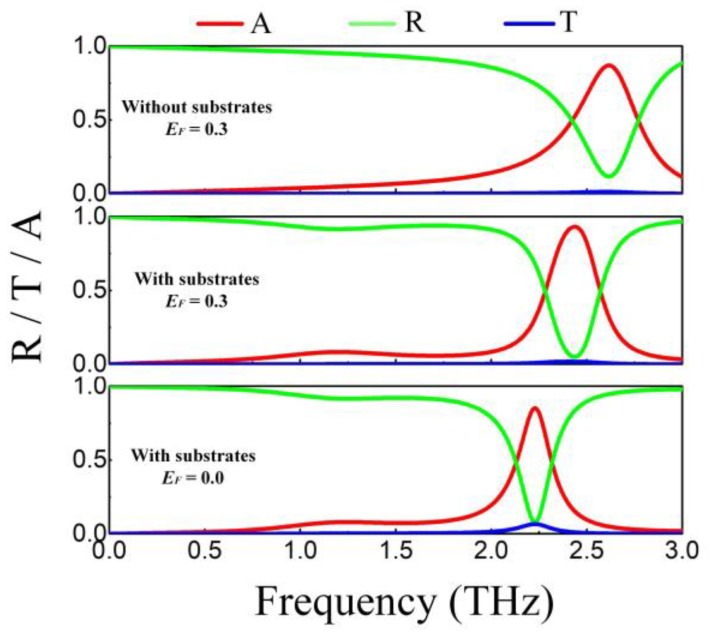
Absorption, reflection and transmission of the frequency-tunable absorber.

The spectrum response of the frequency-tunable absorber with *E_F_* = 0.3 eV is indicated in Figure 7. There is an absorbing point peaking at 2.7 THz. The frequency-tunable absorber is utilized to replace the gold reflector of absorption-controllable absorber when constructing the dual-band absorber. Therefore, the substrates of absorption-controllable absorber are placed on the front layer of frequency-tunable absorber to simulate together. The resonant frequency drops to 2.48 THz with absorption more than 90%. It agrees well with the conclusion of Ref. [42], that attaching substrate slab with finite length to the one side of periodic resonant structure will decrease the resonant frequency. When *E_F_* varies from 0.3 to 0 eV, the resonant position appears red shift, peaking at 2.29 THz. The parallel capacitance and inductance resonance of hybrid structure has a negative correlation between the resonant frequency and total paralleled inductance. The decrease of *E_F_* increases the inductance of graphene, which improves the total paralleled inductance of the structure thus moving the absorbing peak to lower frequency.

**Figure 8:**
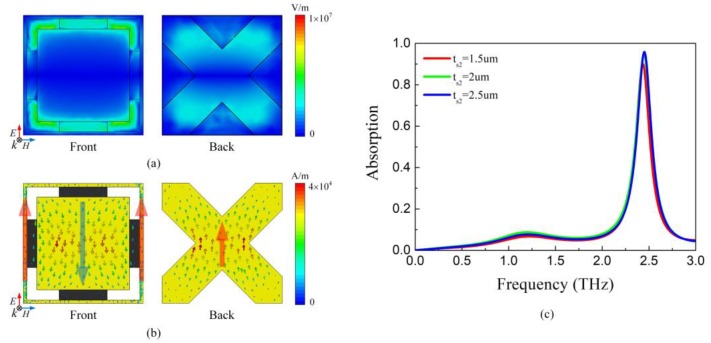
(**a**) The electric field distribution of the narrowband frequency-tunable absorber at 2.48 THz with *E_F_* = 0.3 eV (**b**) The current distribution of the narrowband frequency-tunable absorber at 2.48 THz with *E_F_* = 0.3 eV (**c**) Absorption spectrum of the narrowband frequency-tunable absorber with different *t_s2_* when *E_F_* = 0.3 eV.

The distributions of electric field and surface current on the front layer and back layer are investigated to further explain the absorption mechanism. The absorption peaks at 2.48 THz when *E_F_* = 0.3 eV. As shown in Figure 8a, the electric field focuses on the gap between outer patch and outer grid on the top layer while it gathers in the oblique grid just below the position of square patch on the back layer. The absorption can be attributed to these factors: the stronger resonance between the square patch and the outer grid (on the top layer), the weaker resonance between the square patch and oblique grid (between two layers). The graphene strips in the gap mainly regulate the first factor. In other words, the capacitance of square patch along with the inductance of the outer grid, the oblique grid and the graphene forms the parallel resonance, whose power is dissipated by the graphene and gold. This principle of operation can be further verified by the current distribution displayed in Figure 8b. The current on square patch of the front layer flows from top to bottom while it is inversed on the outer grid and back layer. The anti-parallel currents are triggered by the magnetic resonance between the patches and the grids, consistent with previous research results [43].

In the narrowband frequency-tunable absorber, the absorption spectrum with different spacer thickness *t_s2_* is indicated in Figure 8c. It can be seen that the absorptivity decreases gradually with the increase of *t_s2_*. The decrease of spacer thickness enhances the weak resonance between layers. Therefore, the intense of the top-layer resonance is weakened. As a result, the energy absorbed by graphene reduces as well, thus leading to the drop of absorption.

**Figure 9:**
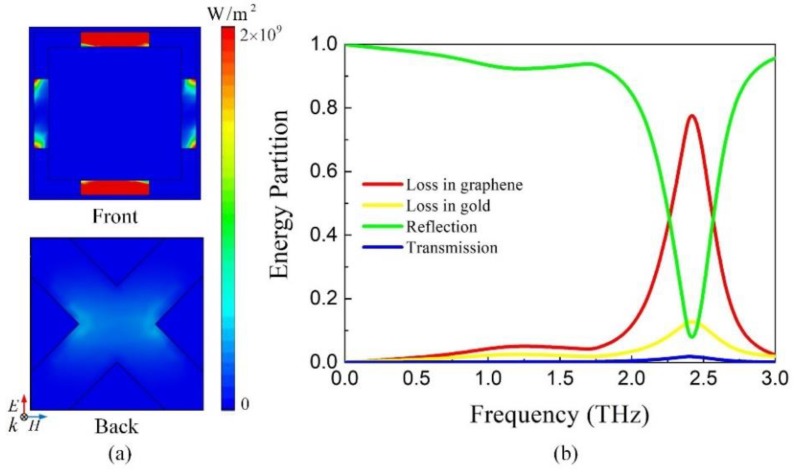
(**a**) The power loss distribution of the narrowband frequency-tunable absorber at 2.48 THz with *E_F_* = 0.3 eV (**b**) The energy partition of the narrowband frequency-tunable absorber at 2.48 THz with *E_F_* = 0.3 eV.

Furthermore, the distributions of power loss are indicated in Figure 9a. It can be seen that most of the energy is dissipated within graphene strips in accordance with the strong resonance on the front layer while a small portion of the energy is consumed in of gold-graphene should be dominant so that the performance of absorber can be tuned by the gate voltage. As shown in Figure 9b, at 2.49 THz, the energy dissipated by graphene reaches 80% and the gold also makes a small partial contribution to the loss of energy. Therefore, the frequency of absorber can be effectively regulated by the change of chemical potential of graphene.

### 3.3. Simulations of Independently Tunable Dual-Band Absorber

**Figure 10:**
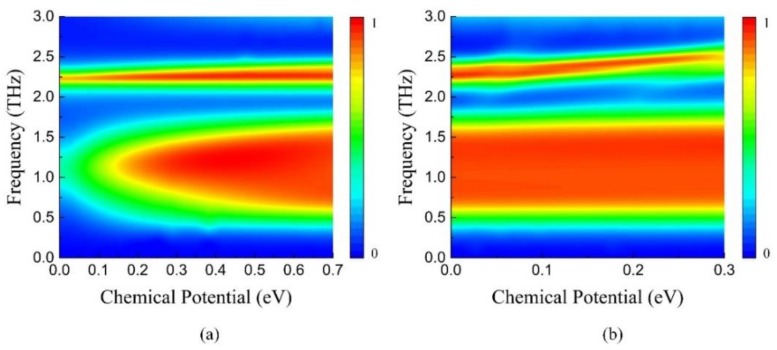
Absorption spectrum of the independently tunable dual-band absorber as a function of frequency and chemical potential (**a**) *E_F1_*: 0–0.7 eV, *E_F2_* = 0 eV (**b**) *E_F1_* = 0.7 eV, *E_F2_*: 0–0.3 eV.

Figure 10a gives the absorption spectrum of the dual-band tunable absorber as a function of the frequency and chemical potential *E_F1_*. When *E_F1_* increases from 0 to 0.7 eV with *E_F2_* = 0 eV, the maximum absorption of *f_1_* (0.72 to 1.6 THz) sustains a continuous growth from 30% to more than 90%. At the same time, the second absorbing peak located at *f_2_* = 2.3 THz maintains the absorption over 90% without frequency shift due to the fixed *E_F2_*. However, as shown in Figure 5a, the absorption of nanostructure graphene improves from 0 to 10% at 2.3 THz with the increase of *E_F1_*, which leads to the gradual expansion of effective operating bandwidth (over 90%) of the second absorbing peak. Figure 10b illustrates the relation between the absorption, frequency and *E_F2_* with *E_F1_* fixed at 0.7 eV. The effective absorbing band from 0.72 to 1.6 THz remains unchanged while the operating frequency of the second peak *f_2_* moves from 2.3 to 2.5 THz, as *E_F2_* grows from 0 to 0.3 eV. During the blue shift of *f_2_*, the effective bandwidth of the absorbing peak becomes narrow slowly, which is attributed to the decreased absorption of nanostructure graphene from 2.3 to 2.5 THz. In brief, the dual-band absorber combines the characteristics of broadband absorption-controllable absorber and narrowband frequency-tunable absorber. The absorption at *f_1_* and the position of *f_2_* can be tuned independently and freely.

**Figure 11:**
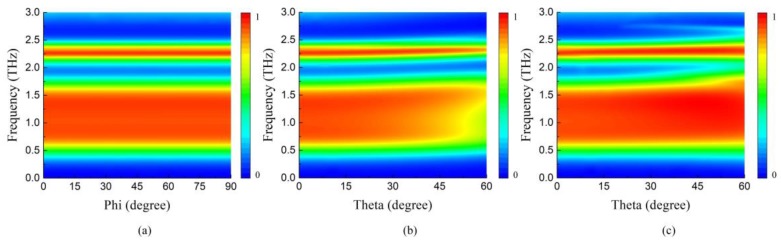
Absorption spectrum of the independently tunable dual-band absorber as a function of frequency and polarization angles/incident angles (**a**) polarization angle *ϕ*: 0°–90°; (**b**) TE, incident angle *θ*: 0°–60° (**c**) TM, incident angle *θ*: 0°–60°.

The independence of absorption with respect to angle and polarization is essential in practical applications. Therefore, the absorption under different incident angles and polarized wave is investigated. Here, the chemical potentials *E_F1_* and *E_F2_* are fixed at 0.7 and 0 eV. Figure 11a depicts the absorption spectra with different polarization angle *ϕ* of incident wave. As *ϕ* varies from 0° to 90°, the absorption spectrum has no change since the C4 symmetry of the structure ensures the robustness. For TE (Transverse electric) and TM (Transverse magnetic) polarized wave under oblique incident angle *θ*, the absorption spectrums are shown in Figure 11b,c, respectively. The absorption at both *f_1_* and *f_2_* remains over 90% up to 50° for TE polarized wave. However, as *θ* continues to rise, there is a decline tendency for the absorption at narrow absorbing peak *f_2_* and the low frequency portion of the broad absorbing band *f_1_*. The absorptions at these regions is triggered by the magnetic resonance on the same layer. With the increase of incident angle for TE waves, the transverse magnetic field decreases gradually, so does the intensity of magnetic resonance, resulting in a drop in absorption. As for TM polarized wave, the magnetic resonance and localized surface plasmon resonance are not affected by the incident angle so that the absorption remains more than 90% over a wide range of incidence angles up to 60°. Besides, the broad absorbing band is expanded to higher frequency when *θ* = 50°, because some parasitic resonances occur and become stronger at large incident angle [13,18]. The incident angle and polarization insensitivity make the proposed absorber a suitable candidate for various applications such as THz detection, sensing and telecommunication.

## 4. Conclusions

A dual-band tunable absorber has been designed and demonstrated theoretically, by cascading two different types of absorber. The first broad absorbing band is produced by the hybridization of different resonances in the nanostructure graphene. By changing the voltage between the graphene and doped Si to control the resonance intensity, the absorption can be continuously tuned from 30% to 90% over 0.72 to 1.6 THz. The second narrow absorbing band is formed by the magnetic resonance of gold-graphene hybrid structure. The absorbing peak with 90% absorptivity can be varied between 2.3 to 2.5 THz via controlling the voltage between hybrid layer and back grid layer. The two operating bands are controlled with different electrode gates and can be independently regulated without interference, thus greatly expanding the scope of its applications. This paper presents the general method of designing broadband absorption-controllable absorber and frequency-tunable absorber. In addition, the integration technique for combining the mentioned reconfiguration capabilities is elaborated, which is also available in other frequency for the scalability.
